# The complete mitochondrial genome of *Anisakis pegreffii* Campana-Rouget & Biocca, 1955, (Nematoda, Chromadorea, Rhabditida, Anisakidae) – clarification of mitogenome sequences of the *Anisakis simplex* species complex

**DOI:** 10.1080/23802359.2017.1318678

**Published:** 2017-04-24

**Authors:** Akinori Yamada, Natsuki Ikeda, Haruka Ono

**Affiliations:** Graduate School of Fisheries and Environmental Sciences, Nagasaki University, Nagasaki, Japan

**Keywords:** *Anisakis pegreffii*, mitochondria, genome, *Anisakis simplex*

## Abstract

The complete mitochondrial genome of *Anisakis pegreffii* (former *A. simplex* A) was determined using the Illumina HiSeq platform. The genome was 14,002 bp in length made up of 36 mitochondrial genes (12 CDSs, 22 tRNAs, and 2 rRNAs). Phylogenetic analysis clarified the mitogenome sequences of the three sibling species of the *A. simplex* species complex, *A. pegreffii*, *A. simplex sensu stricto* and *A. berlandi* (former *A. simplex* C).

Anisakid nematodes are the well-known parasites of marine fish and squids, and can cause anisakiasis (anisakidosis) if they infect humans through raw or undercooked seafood. The representative of the family is the genus *Anisakis,* particularly the *Anisakis simplex s*pecies complex (*A. simplex sensu lato*), which are most commonly involved in human infections (Audicana & Kennedy 2008). Genetic and molecular studies have revealed that *A. simplex s.l.* is made up of the three sibling species, *A. simplex sensu stricto* (Rudolphi 1809, det. Krabbe 1878), *A. pegreffii* Campana-Rouget & Biocca 1955, and *A. berlandi* Mattiucci et al. 2014 (Mattiucci et al. 1997, 2014; Mattiucci & Nascetti 2006, Valentini et al. 2006). In Japan, where the majority of anisakiasis cases are reported (Audicana & Kennedy 2008), the two species *A. simplex s.s.* and *A. pegreffii* are dominantly isolated from marine fishes (Quiazon et al. 2011), while most of the human infections seem to have been caused by the former (Umehara et al. 2007). The mitogenome information of the three sibling species would be useful to develop further molecular markers for their identification and characterization. So far, the complete mitogenome sequences have been reported for the specimens identified as *A. simplex s.l., A. simplex s.s.,* and *A. berlandi* (Figure 1); we determined the mitogenome sequence of *A. pegreffii* and clarified the relationships among the three sibling species of *A. simplex s.l*.

**Figure 1.**
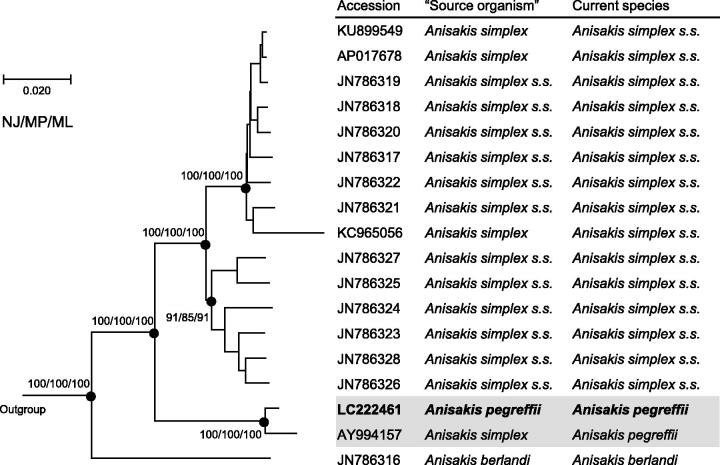
Phylogenetic relationships of the three sibling species of the *A. simplex* species complex (*A. simplex sensu lato*) inferred from the complete mitogenome analysis using NJ (K2P model), MP and ML (GTR + I + G model) methods each with 1000 replicates of bootstrap (100 replicates for ML). There were a total of 13,457 positions in the dataset. The tree shown is an NJ tree, and the ML and MP trees were almost the same topology. Bootstrap support values are shown for the major nodes. The three species, *Pseudoterranova decipiens sensu lato* Germany*, Toxocara canis, Ascaris suum* (accession numbers: KU558723, AP017701, X54253, respectively) were used as outgroup. ‘Source organism’ is that in GenBank.

Specimens (Specimen Voucher: Nagasaki University #Ani160315) were isolated from the viscera of Chub mackerel, *Scomber japonicus*, caught near the Goto Islands off Nagasaki (129°16'E, 33°19'N), where *S. japonicus* are shown to be infected exclusively by *A. pegreffii* (Quiazon et al. 2011). Total DNA of the specimens was extracted, and whole genome sequencing was outsourced to Macrogen (Seoul, South Korea). A total of 87M 101-bp paired-end reads generated by Illumina HiSeq 4000 were assembled using IDBA_UD (Peng et al. 2012). Contigs that agreed with the known *A. simplex s.l.* mitogenomes were further manually assembled based on the depth of coverage, resulting in a circular mitogenome. The mitogenome was annotated with MITOS (Bernt et al. 2012) followed by manual validation of the coding regions using the reference genomes. Phylogenetic analyses were conducted using MEGA7 (Kumar et al. 2016).

The complete mitogenome of *A. pegreffii* was 14,002 bp in length (accession number: LC222461), and contained 36 mitochondrial genes (12 CDSs, 22 tRNAs, and 2 rRNAs) in the same order as the other *Anisakis* mitogenomes. The constructed phylogenetic tree showed the genome of *A. pegreffii* was closely related to that of *A. simplex s.l.* (accession number: AY994157, Kim et al. 2006) (Figure 1), which was further identified to belong to *A. pegreffii* based on an additional analysis of COII gene sequences of *Anisakis* species. The three sibling species were distinctly separated from each other and also strongly supported by bootstrap values. The mitogenome sequences will facilitate molecular species identification as well as genetic population analysis. Actually, Mattiucci et al. (2013) pointed out the possible association between mitochondrial haplotypes and pathogenic features of *A. pegreffii*-mediated anisakiasis.
